# Extracorporeal Life Support in a Severe Blunt Chest Trauma with Cardiac Rupture

**DOI:** 10.1155/2013/136542

**Published:** 2013-09-30

**Authors:** Launey Yoann, Flecher Erwan, Nesseler Nicolas, Malledant Yannick, Seguin Philippe

**Affiliations:** ^1^Anesthesiology, Critical Care and Emergencies, Rennes University Hospital, 35000 Rennes, France; ^2^Anesthesiology, Critical Care, Emergencies, and SAMU (Service d'Aide Médicale Urgente), Rennes 1 University, Inserm U991, Centre Hospitalier Universitaire (CHU) Pontchaillou, 35000 Rennes, France; ^3^Department of Cardiovascular and Thoracic Surgery, Rennes University Hospital, 35000 Rennes, France

## Abstract

This report presents a case of severe blunt chest trauma secondary to a horse riding accident with resultant free-wall rupture of the left ventricle in association with severe lung contusion. We describe the initial surgical and medical management of the cardiac rupture which was associated with a massive haemoptysis due to severe lung trauma. Extra corporeal membrane oxygenation (ECMO) support was initiated and allowed both the acute heart and lung failure to recover. We discuss the successful use and pitfalls of ECMO techniques which are sparsely described in such severe combined cardiac and thoracic trauma.

## 1. Introduction

Traumatic cardiac rupture rarely complicates blunt chest trauma but is almost always fatal due to sudden and massive bleeding. A rare proportion of victims who reach the hospital alive benefits from emergency cardiac surgery. However, heart rupture is often associated with severe heart failure in the postoperative period often resulting in death [1]. Extra corporeal life support (ECLS) has been utilized for the last 15 years, and its indications are now rapidly spreading [2]. With permission from our local Institutional Review Board, we are publishing an original case report of heart and lung traumatic injuries requiring urgent surgery and ECLS implantation during the surgical procedure and the postoperative course.

## 2. Case Report

A 37-year-old woman was admitted to our emergency department for thoracic blunt trauma caused by chest trampling after a fall from a horse. Her medical history includes a Guillain-Barré syndrome without sequelae and a previous traumatic brain injury responsible of rare mnesic disorders. At the initial evaluation, the patient was conscious without any motor deficit. Clinical examination revealed severe hypoxia with pulse oximetry (SpO_2_) at 89% increasing to 98% breathing under O_2_ at 15 liters/min, a tachycardia of 120 bpm without hypotension (arterial blood pressure = 110/78 mmHg). The patient was complaining, however, of dorsal and left-sided chest pain. At admission, the respiratory status promptly deteriorated with increased hypoxemia. Initial chest X-ray revealed a left pneumothorax with bilateral lung contusions. Despite chest tube insertion, respiratory failure worsened, and trachea was intubated with immediate bradycardia, ventricular fibrillation, and finally asystole. Cardiopulmonary resuscitation with chest compression associated with intravenous (iv) adrenaline (total amount = 2.6 mg) was necessary for recovery of an effective circulatory activity. FAST-ultrasound scan identified pericardial tamponade and a small intraperitoneal effusion. The initial blood tests revealed an acute fibrinolysis (prothrombin rate < 10%, fibrinogen < 0.1 g/L, and D-dimers > 45 *μ*g/mL with normal platelet count) and an anemia with hemoglobin level of 74 g/L. Under mechanical ventilation, arterial blood analysis revealed acidosis with a pH of 6.99, PaCO_2_ of 57 mmHg, bicarbonate of 16 mmol/l, and PaO_2_/FiO_2_ ratio of 114 mmHg.

**Figure 1:**
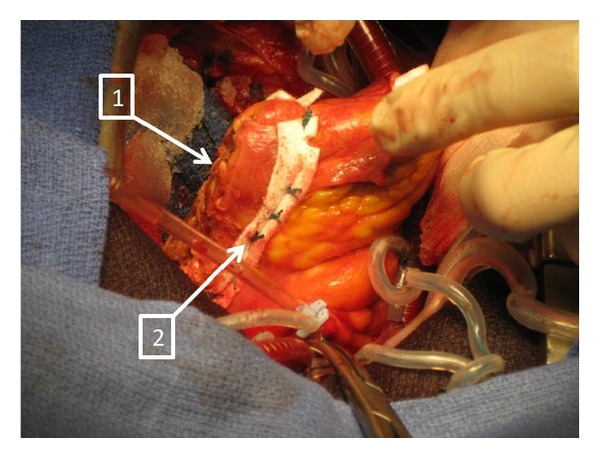
Arrows entitled (1) show the left-ventricle rupture stitched during cardiac surgery with the ECMO device. Arrows entitled (2) depicts two strips of felt placed lengthwise on the epicardial surface and used to support the closure of the rupture from outside the myocardium, using horizontal mattress sutures of 2/0 polyester.

**Figure 2:**
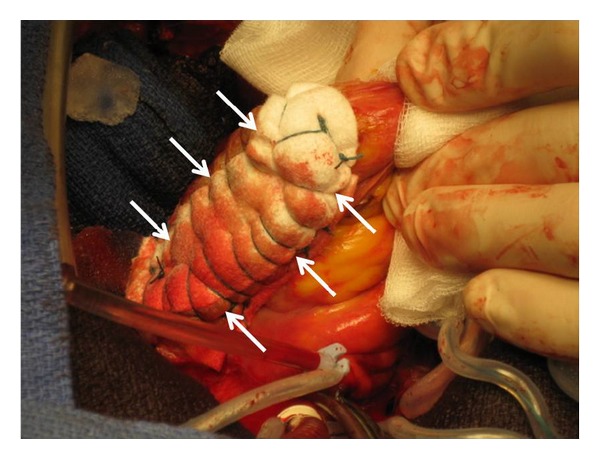
Arrows delimit the insertion of the felt patch used for the closure of the left-ventricle rupture.

As blood transfusion was initiated the patient was transferred to the operating room. A massive hemopericardium was first evacuated through median sternotomy, revealing active bleeding from a left free-wall ventricle rupture with a myocardial tear measuring 3 cm wide, located close to the circumflex coronary artery. In order to repair the ventricular rupture, emergent extracorporeal circulation was established between the ascending aorta and the right atrium after systemic anticoagulation with 20,000 units (300 ui/kg) iv of heparin. Two strips of felt were then placed lengthwise on the epicardial surface and were used to support the closure of the rupture from outside the myocardium, using multiple horizontal mattress sutures of 2/0 polyesters (Figures 1 and 2). Surgical glue was then added to obtain perfect haemostasis. After weaning from cardiopulmonary bypass, significant lung haemorrhage from the left lower lobe (confirmed with preoperative bronchial endoscopy) was found to be responsible for massive haemoptysis and severe preoperative hypoxemia (SpO_2_ = 80%). Via median sternotomy, the lung contusion was evident, with significant left lower lobe destruction responsible for the intrabronchial bleeding. At that time, the platelet count was 92 giga/L, prothrombin ratio (PT) was 52%, activated partial thromboplastin time (APTT) ratio was increased by 2.1 times normal (71 versus 34 for control), and the fibrinogen was 2.31 g/L. As it was deemed technically impossible to perform the lobar resection for haemostasis through the sternotomy and taking into account the hemodynamic and respiratory instability, the surgeon decided to first establish venoarterial extracorporeal membrane oxygenation (ECMO) between the right femoral vein and artery with heparin-coated cannulas. Following this, a standard posterolateral left thoracotomy was performed and permitted the performance of a left-lower lobectomy with immediate resolution of the intrabronchial bleeding. During pre- and postoperative periods, a total of 17 units of packed red blood cells, 8 fresh frozen plasma units, 2 platelets units, and 6 g of fibrinogen were administered. During the immediate postoperative period, the ECMO support was maintained, and the patient was transferred to our intensive care unit (ICU). A full-body scan was performed to evaluate other potential concurrent injuries and revealed a peritoneal effusion and a splenic contrast leak. The decision was made to perform an emergency laparotomy and splenectomy on the same day, still under ECMO support. No other intra-abdominal injury was found. Inotropic support (dobutamine) was used during both surgical procedures and ceased in the early stage of ICU recovery. The ECMO support was continued due to persistence of left heart failure.

Thereafter, the ventricular function slowly improved. Initially severe myocardium dysfunction was observed (left ventricular ejection fraction (LVEF) of 25%, as measured by transoesophageal echography) with associated grade 3/4 mitral regurgitation caused by ischemic restriction of the posterior mitral valve leaflet. At day 8, the LVEF was found to be 50% associated with 2/4 mitral regurgitation allowing ECMO removal. During this period, acute renal failure with anuria required continuous venovenous hemofiltration for three weeks, at which point a complete renal recovery was made. The weaning of sedative medication commenced on day 4, and neurological evaluation revealed slight psychomotor weakness and behaviour disturbances. Mechanical ventilation weaning was delayed on day 18 due to *Enterobacter cloacae* pneumonia. This was treated by cefepime and amikacin administration for 8 and 3 days, respectively. A ruptured pseudoaneurysm developed on the insertion site of ECMO cannula required emergent surgery on day 26. The evolution was then complicated by acute respiratory failure of multifactorial origins (including muscular weakness, bronchitis, and pulmonary oedema) requiring a repeated mechanical ventilation period of 8 days (from day 30 to day 37), to achieve a definitive weaning at day 37.

The patient was transferred to the cardiology ward on day 40 and discharged from hospital on day 50 with a complete neurologic recovery and good cardiac function. Her medication included aspirin, a beta-blocker, and an ACE-inhibitor. At 3-month followup, the clinical condition was satisfactory without any dyspnea (class I NYHA), and Doppler-echocardiographic control showed a left-ventricle ejection fraction of 60% with lateral hypokinesis and residual grade 2/4 mitral regurgitation. 

## 3. Discussion

Cardiac rupture is rarely observed mainly because most patients die prior to reach the hospital as it was highlighted in an autopsy study performed on patients with blunt chest trauma [3]. Nevertheless, in a recent retrospective study focusing on blunt chest trauma, cardiac rupture incidence was found in only 1/2400 patients, but with a very high mortality rate of 89.2% [4]. Traumatic free-wall heart rupture management is a difficult medical and surgical challenge. Survival depends on the rapidity of the patient transfer to a cardiac surgery center. Active bleeding, frequently associated with tamponade, makes the blood pressure initially unstable. Secondary myocardial contusion and myocardial stunning complicate the surgical repair and result in an uncertain postoperative recovery. In the present case, the circumflex coronary artery was located adjacent to the ventricular rupture, and the repair resulted in the occlusion of one of the obtuse marginal coronary arteries as has previously been reported [5]. Myocardial contusion and iatrogenic lateral infarction due to the repair resulted in postoperative left ventricular failure. In our case, the clinical situation deteriorated further due to massive hemoptysis which contributed to hemodynamic disturbances and poor oxygenation. Left lung surgery was impossible through the sternotomy, and the patient required to be repositioned to perform the standard posterolateral left thoracotomy. In this case, venoarterial ECMO support seemed the most appropriated strategy to manage the pitfalls of LV dysfunction and hypoxia prior to the lobectomy being performed. The ECMO was used during the lung resection to facilitate the surgery in terms of ventilation and oxygenation and to support associated unstable blood pressure. Furthermore, ECMO support allowed the maintenance of normothermia, as well as rapid and massive administration of fluids and blood products into the circuit of the pump as needed. In the postoperative course, ECMO support allowed maintaining good systemic perfusion and decreasing the doses of vasopressor drugs despite severe cardiac dysfunction. The ECMO partially unloaded the left ventricle with a view to optimizing myocardium recovery and to limit pulmonary injury. The patient transport for CT-scan and even abdominal surgery was realized under ECMO support without bleeding complication or thrombosis. ECMO pretreated heparinized tubing often allows a lower level of anticoagulation, especially when the pump speed is high. 

In cases of cardiac injuries, few data exist on the management of free-wall cardiac rupture. The diagnosis is based on clinical status associated with a systematic echocardiographic assessment in the context of trauma [6]. Surgical treatment is mandatory, but no guidelines are defined. The use of ECMO support has already been described in a right ventricular rupture due to blast injury [7] or to manage severe combined pulmonary and myocardial contusion [8]. Indeed, myocardial contusion in chest trauma may require high dose of vasoactive drugs and time to recover. Surgical sutures through an injured myocardium are fragile and may lacerate the muscle if the wall tension is too high. Partial unloading of the left ventricle using ECMO might play a role in those fragile surgical repairs. Moreover, such temporary mechanical circulatory support supplies the transiently low native cardiac output and allows decreasing the dose of vasoactive drugs while maintaining good organs perfusion and function. 

To conclude, the present case highlights that ECMO support is a helpful and useful tool to manage the surgical treatment of cardiac ruptured and/or lung injuries induced by trauma and is also of benefit in the postoperative recovery.
